# PD-L1 in Merkel Cell Carcinoma: From Tumor Microenvironment to Pathological Interpretation

**DOI:** 10.3390/ijms27198460

**Published:** 2026-09-23

**Authors:** Ludovica Pepe, Vincenzo Fiorentino, Mario Della Mura, Alessandro Rizzo, Mariacarmela Santarpia, Antonio Ieni, Gerardo Cazzato, Maria Lentini

**Affiliations:** 1 PhD Program in Translational Molecular Medicine and Surgery, Department of Biomedical Sciences, Dental Sciences and Morpho-Functional Imaging, University of Messina, 98125 Messina, Italy; ludovica.pepe@studenti.unime.it; 2 Department of Human Pathology of Adults and Developmental Age “Gaetano Barresi”, Division of Pathology, University of Messina, 98125 Messina, Italy; 3 Section of Molecular Pathology, Department of Precision and Regenerative Medicine and Ionian Area (DiMePRe-J), University of Bari “Aldo Moro”, 70124 Bari, Italy; 4 S.S.D. C.O.r.O. Bed Management Presa in Carico, TDM, IRCCS Istituto Tumori “Giovanni Paolo II”, 70124 Bari, Italy; rizzo.alessandro179@gmail.com; 5 Medical Oncology Unit, Department of Human Pathology “G. Barresi”, University of Messina, 98125 Messina, Italy; mariacarmela.santarpia@unime.it

**Keywords:** Merkel cell carcinoma, PD-L1, PD-1, Merkel cell polyomavirus, immunohistochemistry, tumor microenvironment, immune checkpoint inhibitors, pathology reporting

## Abstract

Merkel cell carcinoma (MCC) is a rare, aggressive cutaneous neuroendocrine carcinoma in which Merkel cell polyomavirus (MCPyV) oncoproteins and ultraviolet-induced mutations provide distinct sources of tumor immunogenicity. This narrative review examines the biological determinants, immunohistochemical assessment, and prognostic and therapeutic relevance of programmed death ligand 1 (PD-L1) in MCC, linking tumor immunobiology to pathological interpretation. PD-L1 expression on tumor and immune cells is often concentrated at tumor–immune interfaces, consistent with interferon-γ-driven adaptive immune resistance. Reported associations with survival and treatment response vary across studies, partly reflecting differences in tissue sampling, assays, scoring compartments, and treatment context. Immune checkpoint blockade can produce durable responses in both PD-L1-positive and PD-L1-negative tumors, while baseline PD-L1 expression alone is not a validated criterion for treatment selection. Resistance mechanisms and emerging biomarkers are discussed alongside viral status, T-cell architecture, antigen-presentation defects, and alternative immune checkpoints. A reporting framework should emphasize specimen characteristics, assay documentation, separate assessment of PD-L1 expression in tumor and immune cells, and spatial heterogeneity. Interpreting PD-L1 within this biological and pathological context supports informative reporting and highlights the potential of integrated spatial biomarkers to improve understanding of immune responsiveness in MCC.

## 1. Introduction

Merkel cell carcinoma (MCC) is a rare and highly aggressive primary cutaneous neuroendocrine carcinoma first described by Toker in 1972 [1]. Its incidence varies geographically, is highest in populations with substantial ultraviolet (UV) exposure, and increases sharply with age; men are affected more often than women [2,3,4]. Advanced age, chronic UV exposure, and impaired immune surveillance are central epidemiologic features. Immunosuppression related to solid-organ transplantation, HIV infection, hematologic malignancy, or immunosuppressive treatment is present in a clinically important minority of patients and is associated with poorer outcomes [5]. Although the historical term arose from a presumed relationship with normal Merkel cells, the cell of origin remains unsettled, and contemporary models favor lineage plasticity or transformation of a permissive cutaneous precursor rather than simple derivation from a mature Merkel cell [6,7,8,9,10].

Clinically, MCC usually presents as a rapidly enlarging, painless, firm, red-to-violaceous nodule on sun-exposed skin [11]. Histologically, it is composed of dermal sheets, nests, or trabeculae of small round blue cells with scant cytoplasm, finely granular chromatin, brisk mitotic activity, and frequent apoptosis or necrosis [12]. Diagnosis requires correlation with immunohistochemistry: CK20 commonly shows a paranuclear dot-like pattern, neuroendocrine markers are expressed, and SATB2 may support the diagnosis, whereas TTF-1, CK7, CD45, and melanocytic markers help exclude major mimics [13,14]. CM2B4 immunohistochemistry can identify MCPyV large T antigen in virus-associated tumors, although molecular and serologic approaches address different biological questions [15].

MCC has a marked propensity for regional and distant spread, and stage at diagnosis remains the dominant clinical prognostic factor. Contemporary population data confirm substantial mortality, while retrospective series show that most recurrences occur within the first two years and that MCPyV-negative tumors may follow a more aggressive course [16,17,18]. Management of localized disease is based on complete excision, sentinel lymph node evaluation in clinically node-negative patients, and risk-adapted radiotherapy according to MCC-specific guidance [19]. Cytotoxic chemotherapy can induce rapid responses in advanced disease, but these are usually short-lived [20]. By contrast, PD-1/PD-L1 blockade can produce durable tumor control and has transformed systemic treatment [5,19,21]. This review therefore examines what PD-L1 expression means biologically, how it can be assessed by pathologists, what the conflicting prognostic and predictive data actually support, and where the limits of current clinical interpretation should be placed.

## 2. Review Methodology

This is a structured narrative review. PubMed/MEDLINE and Europe PMC searches covered database inception through 4 September 2026, combining “Merkel cell carcinoma” or “Merkel cell cancer” with PD-L1/PD-1, immunohistochemistry, tumor microenvironment, prognosis, treatment prediction, checkpoint blockade, resistance, human leukocyte antigen (HLA)/antigen presentation, and spatial pathology. Agent- and trial-specific searches and reference-list screening supplemented this approach. Primary publications and available supplements were checked for assay methods, evaluable denominators, and endpoint definitions. Regulatory indications were assessed on 6 September 2026, and guideline recommendations were checked against the cited versions.

Sources included peer-reviewed MCC clinical, pathology, translational, and laboratory studies; disease-focused syntheses; and authoritative guidance, regulatory, and trial reports. The bibliography was checked for duplicates using DOI, PMID/PMCID, and title. Sources assessed only through their abstracts were identified as such. Technical details not specified in the full texts or supplementary materials examined were reported as unspecified.

This review draws on 129 sources, including 83 peer-reviewed original MCC publications. These counts refer to publications rather than independent patient cohorts. Evidence was synthesized narratively, focusing on differences in sampling, assays, scoring compartments, and clinical findings.

## 3. Immunobiological Basis of PD-L1 Expression in MCC

### 3.1. MCPyV-Positive and UV-Associated Oncogenic Pathways

Most MCCs follow one of two major oncogenic routes. MCPyV-positive tumors show clonal viral integration, sustained expression of small T antigen and a truncated large T antigen that retains retinoblastoma protein (RB)-binding activity, low tumor mutational burden, and relatively few recurrent somatic alterations [22,23,24,25]. Their antigenic substrate is largely provided by non-self viral oncoproteins. MCPyV-negative tumors instead show a high UV mutational burden, characteristic C > T transitions at dipyrimidine sites and CC > TT tandem substitutions, and frequent disruption of *TP53*, *RB1*, chromatin-regulatory genes, and DNA-damage pathways [26,27,28]. This UV-associated route contrasts with the direct expression of MCPyV T antigens in virus-positive tumor cells [29].

Despite their distinct etiologies, both groups can recruit tumor-reactive lymphocytes. MCPyV-specific T-cell responses are detectable in virus-positive disease [30,31,32,33], whereas UV-associated mutation-derived neoantigens provide a distinct immunogenic substrate in virus-negative tumors [26,27,28]. Intratumoral CD8+ T-cell infiltration has been associated with improved outcome independently of stage in retrospective cohorts [34,35]. The recognized, although uncommon, phenomenon of spontaneous regression provides additional clinical evidence that MCC can be controlled by host immunity [36]. Immunogenicity alone, however, is insufficient: many tumors remain poorly infiltrated, and even inflamed lesions can establish multiple layers of local immune suppression.

### 3.2. Tumor Microenvironment and Adaptive Immune Resistance

The MCC tumor microenvironment (TME) contains variable proportions of cytotoxic and helper T-cells, natural killer cells, regulatory T-cells, dendritic cells, and tumor-associated macrophages [37,38,39,40]. CD8+ T-cells represent a major effector population, but their density, intratumoral penetration, differentiation state, and proximity to tumor cells may be more informative than simple presence or absence. CD163+ macrophages, regulatory T-cells, and selected dendritic-cell populations can contribute to a suppressive niche, while chronic antigen exposure is associated with co-expression of inhibitory receptors such as PD-1, TIM-3, LAG-3, TIGIT, and CTLA-4 [37,40,41,42,43].

PD-L1 expression in MCC is frequently interpreted as being consistent with adaptive immune resistance. Tumor-reactive lymphocytes can release interferon-γ (IFN-γ), activating JAK/STAT signaling in nearby tumor and immune cells and inducing PD-L1 at tumor–immune interfaces. Accordingly, staining may be strongest at an invasive margin or adjacent to dense lymphohistiocytic aggregates rather than uniformly distributed throughout the neoplasm [39,40]. Figure 1 summarizes this conceptual model; it depicts PD-L1 as a spatial signal of an ongoing immune interaction rather than as a binary tumor-cell attribute.

### 3.3. PD-1/PD-L1 Signaling and Therapeutic Susceptibility

PD-1 and PD-L1 expression has been investigated in MCC and related cutaneous-tumor contexts [37,40,44,45]. Mechanistically, engagement of PD-1 by PD-L1 or PD-L2 recruits inhibitory phosphatases and attenuates T-cell receptor signaling, proliferation, cytokine production, and cytotoxic function [46]. In MCC, PD-L1 can be expressed by tumor cells, macrophages, dendritic cells, and other immune cells, and its distribution is closely linked to inflammatory architecture [39,44,47,48,49]. Martins et al. identified PD-1 expression by MCC cells themselves and reported tumor-intrinsic proliferative signaling through mTOR, PRAS40, mitochondrial respiration, and reactive oxygen species; however, their mechanistic observation does not establish a clinical tissue biomarker [50].

The clinical activity of checkpoint blockade provides the strongest evidence that this pathway is therapeutically relevant. Avelumab, pembrolizumab, retifanlimab, and nivolumab-based regimens have produced objective and sometimes durable responses in advanced MCC [51,52,53,54,55]. MCC-specific correlative studies have linked response to clonally expanded or central-memory tumor-reactive T-cell states and to tertiary lymphoid structures [56,57,58]. Experimental work in chronic viral infection in mice identified stem-like PD-1+TCF1+ CD8+ T-cell populations that expand after PD-1 blockade [59]. However, these data only provide mechanistic context and do not establish PD-1 and/or PD-L1 as MCC-validated tissue biomarkers. Together, these findings support the central interpretive point of this review: the therapeutic relevance of the PD-1/PD-L1 pathway does not make baseline PD-L1 immunohistochemistry a validated stand-alone predictor of benefit.

## 4. Pathological Assessment of PD-L1 in MCC

### 4.1. What Is Directly Supported by MCC Tissue Studies?

MCC-specific tissue studies show that PD-L1 can be expressed by tumor and immune cells, with widely variable prevalence because specimens, clones, platforms, compartments, thresholds, and definitions of positivity differ [39,40,44,48,60,61]. Additional direct series consistently demonstrate the importance of compartment and sampling: peripheral or interface-predominant tumor-cell staining, PD-L1-positive myeloid-cell rims, and heterogeneous tumor-cell positivity have been reported, while clinical associations range from favorable to null [62,63,64,65,66,67,68,69,70,71]. However, these works differ substantially in cohort composition, specimen type, scoring method, and endpoint.

Integrated biomarker studies further place PD-L1 within a broader immune state. Glucose-6-phosphate dehydrogenase expression showed an inverse association with PD-L1 and immune activity [72]; *PDCD1* promoter methylation—which concerns the PD-1 receptor rather than the PD-L1/CD274 ligand—has been investigated as a distinct immune variable [73]; and MCPyV viral load and CD8+ infiltration have been assessed in relation to outcome [74]. None of these variables is part of a clinically validated routine MCC panel.

Spatial studies show why small biopsies and isolated fields may be misleading. In the Giraldo pembrolizumab cohort, 26 pretreatment specimens were studied, with PD-L1 immunohistochemistry available in 25 and PD-1 immunofluorescence in 16. Quantitative density and close PD-1/PD-L1 cellular proximity, rather than a simple binary label, were associated with response [75]. Spassova’s separate immune-profiling analysis found a shorter mean CD8+ T-cell–tumor-cell distance in patients with disease control than in those with progression (13.24 versus 22.00 μm; *p* = 0.009). Samples with CD8+ cells below 1% of total cells were excluded from that distance analysis; this was a CD8-based eligibility criterion, not a PD-L1 positivity cutoff [56]. T-cell architecture and repertoire diversity provide additional, distinct immune-context information [58].

Bénigni’s 58-primary-tumor series used serial whole sections, with clone 22C3 (Dako, 1:50) on Ventana BenchMark Ultra and OptiView DAB detection with signal amplification. Eleven of 58 tumors met the whole-slide tumor-cell threshold of ≥1%, while focal positive fields were identified in 22 of the remaining 47 whole-slide-negative tumors [60]. This within-tumor comparison demonstrates sampling dependence. No patient received PD-1/PD-L1 inhibitors, and treatment-response or prognostic validation was not performed [60].

Table 1 summarizes the main findings on PD-L1 expression in MCC and their implications for pathological assessment and clinical interpretation.

### 4.2. Cross-Tumor Biological and Analytical Evidence: Useful Context, Not MCC Guidance

PD-L1 regulation in other cancers involves DNA-damage signaling, PI3K/AKT/mTOR and MAPK activity, transcriptional and epigenetic control, mRNA stability, and post-translational modification or trafficking [82,83]. Observations from other neoplasms can provide biological context and suggest molecular pathways and mechanisms possibly involved in MCC immune escape [84,85,86,87].

The same boundary applies to assay harmonization. Large comparative programs, mainly in non-small cell lung cancer, indicate that 22C3, 28-8, and SP263 often show broadly similar tumor-cell staining, whereas SP142 tends to identify fewer positive tumor cells and 73-10 may be more sensitive [88,89,90,91]. These studies do not validate interchangeability in MCC, especially for immune-cell scoring or spatial interpretation. Table 2 summarizes the comparative analytical characteristics of the main PD-L1 assays studied in other tumor types, alongside considerations for their application to MCC.

### 4.3. Scoring and Descriptive Reporting

The tumor proportion score (TPS), immune-cell (IC) algorithms, and combined positive score (CPS) were developed for specific drug-tumor-assay contexts. TPS records the percentage of viable tumor cells with partial or complete membranous staining; CPS incorporates PD-L1-positive tumor cells, lymphocytes, and macrophages relative to the number of viable tumor cells; IC algorithms quantify immune-cell staining according to an assay-specific denominator [88,89,90,91]. None of these systems has been clinically validated as the required scoring method for MCC, and cutoffs established in other malignancies should not be imported into MCC without validation.

When PD-L1 is assessed in MCC, a continuous and compartment-specific description is more defensible than an unsupported positive/negative label. The report should identify the specimen and anatomical site, biopsy or resection status, primary or metastatic site, treatment status, clone, platform, protocol, technical adequacy, percentage of membranous tumor-cell staining, immune-cell staining if evaluated, and spatial pattern. Focality, interface enrichment, necrosis, crush artifact, limited viable tumor, and discordance between primary and metastatic or pre- and post-treatment specimens should be stated. Adequacy criteria and minimum tumor-cell counts must follow the assay or study protocol being used; there is no universal MCC-specific minimum or threshold.

## 5. Prognostic and Predictive Relevance

### 5.1. Prognostic Associations in Observational Tissue Cohorts

Prognostic and predictive questions must be separated. A prognostic marker is associated with outcome, whereas a predictive marker identifies differential benefit from a treatment. Lipson et al. associated absence of tumor-cell PD-L1 with worse overall survival in 49 patients after adjustment for age and anatomical site (hazard ratio 3.12, 95% confidence interval 1.28–7.61; *p* = 0.012) [39].

Guénolé et al. included 77 non-metastatic patients treated with surgery with or without radiotherapy, none receiving PD-1/PD-L1 inhibitors; primary-tumor tissue was available for immunohistochemistry in 58. Using clone 22C3 on Ventana BenchMark Ultra, with a ≥1% threshold in each compartment, 11/58 tumors were tumor-cell positive and 40/58 were immune-cell positive. The study reported a favorable univariate tumor-cell association with overall survival (hazard ratio 0.39, 95% confidence interval 0.18–0.83), which was not retained after adjustment (0.82, 0.31–2.15; *p* = 0.69). In contrast, immune-cell positivity remained independently associated with overall survival (0.43, 0.20–0.79; *p* = 0.006), regional control (*p* = 0.012), and distant metastasis-free survival (*p* = 0.003) [68].

Immune-cell positivity was also associated with better disease-specific survival in Toberer’s small primary-tumor cohort, without multivariable validation [70]. Comut et al. reported an independent association between CPS < 1 and shorter overall survival in a 35-case multicenter series, but the PD-L1 clone and the PD-L1-specific evaluable denominator were not retrievable at the time of our search [71].

Overall, prognostic findings are inconsistent: some cohorts showed no significant association with PD-L1, whereas associations in others were not retained after multivariable adjustment. Hanna et al. studied 78 patients not treated with immune checkpoint inhibitors, with PD-L1 evaluable in 67; the overall-survival association was not significant (hazard ratio 1.26, 95% confidence interval 0.46–3.45; *p* = 0.60) [44]. Feldmeyer, Behr, and Richter likewise found no PD-L1-specific survival association [38,62,65]. In Donizy’s 132-primary-tumor study, the favorable association for tumor-cell PD-L1 > 1% did not persist after multivariable adjustment [48]; Naseri, Acikalin, Öğüt, and Siqueira also reported no independent PD-L1 survival effect [61,66,67,69]. Iwasaki found no PD-L1 survival correlation, whereas greater infiltration of cells with low-intensity FOXP3 expression at the invasive front was independently associated with favorable disease-specific survival [41]. Wehkamp reported exploratory adverse-outcome trends [64]. These results should not be treated as measurements of one uniform biomarker: for example, Lipson used a ≥5% tumor-cell threshold, Hanna used an H-score > 0 on triplicate tissue microarrays, and Guénolé’s independent association concerned immune cells. Stage, treatment, viral status, sampling, endpoint, and model adjustment provide additional sources of discordance. Intratumoral CD8+ infiltration has shown a more consistent survival association across independent cohorts [34,35,74].

Abdollahi et al. reviewed 24 studies across skin neoplasms and listed four MCC reports, but their MCC overall-survival forest plot contains only Guénolé’s univariate tumor-cell contrast (≥1% versus <1%; hazard ratio 0.39, 95% confidence interval 0.18–0.83) [92]. This is not a multicohort MCC estimate or independent confirmation, and the source tumor-cell association did not persist after adjustment [68,92].

### 5.2. Prediction of Response to PD-1/PD-L1 Blockade

Prospective single-arm trials show that numerical enrichment and treatment selection are different questions. In first-line JAVELIN Merkel 200, PD-L1 was assessable in 108 of 116 patients using the 73-10 assay and a tumor-cell threshold of ≥1%. Objective response rates were 61.9% (95% confidence interval 38.4–81.9%) in 21 positive patients and 33.3% (23.6–44.3%) in 87 negative patients [52]. Thus, responses were numerically enriched among positives but remained substantial among negatives. In CITN-09/KEYNOTE-017, PD-L1 was assessed with clone 22C3 and a ≥1% compartment-specific threshold in 47 of 50 pretreatment specimens; the response-correlation analysis included 46 patients and found no association. Tumor-cell positivity showed nonsignificant trends toward improved overall and progression-free survival (*p* = 0.057 and *p* = 0.128, respectively) [51]. These within-study results do not establish a validated exclusion criterion, and differences in assays, sampling, and endpoints prevent attributing their discordance to the choice of avelumab versus pembrolizumab.

Giraldo’s exploratory analysis, nested within the initial CITN-09/KEYNOTE-017 cohort, associated quantitative PD-L1 density and PD-1/PD-L1 proximity with pembrolizumab response, without validating a cutoff [75]. It should not be regarded as independent clinical replication of the parent trial [51,75,76]. In Spassova’s 114-patient cohort, tumor PD-L1 status was observed in only 38 patients. A Bayesian ordinal best-overall-response model used multiple imputation rather than complete-case restriction and found no relevant PD-L1 association; extensive missingness limits the strength of that negative inference. Clone and cutoff were not specified by the authors [56]. Separate multiplex panels, which did not include PD-L1, studied 21 patients and associated T-cell phenotype and proximity with disease control, including stable disease [56]. Forty-one clinical patients overlapped with the earlier report [58]. The two studies therefore cannot be counted as fully independent cohorts. Responses in both PD-L1 groups in pivotal trials support avoiding PD-L1-based exclusion from treatment [51,52,76,77].

Schielke et al. retrospectively evaluated 39 patients with advanced MCC using pretreatment 28-8 pharmDx staining and a tumor-cell threshold of ≥1%. PD-L1-positive tumors had better adjusted overall survival (hazard ratio 0.35, 95% confidence interval 0.14–0.90; *p* = 0.03). Objective responses occurred in 10/23 positive and 4/16 negative cases, without a significant between-group difference [93]. This small, single-center, nonrandomized study included different agents and treatment lines. The favorable survival association does not establish a treatment-selection rule. An outcome association among treated patients is not, by itself, evidence of differential therapeutic benefit; predictive validation requires an appropriate comparator or treatment-by-biomarker analysis and independent confirmation. Table 3 compares selected studies of PD-L1 in MCC according to sample characteristics, assay methods, scoring criteria, clinical findings, and study limitations.

Taken together, current data support PD-L1 as a biologically informative component of an inflamed tumor microenvironment, but not as a validated independent prognostic classifier or a stand-alone predictive biomarker. Favorable associations recur in some cohorts, particularly when immune-cell or combined scoring captures active host immunity, whereas other studies are null or lose significance after multivariable adjustment. A negative result cannot be interpreted as evidence of treatment futility, while a positive result does not guarantee response. Any apparent association should be contextualized by stage, treatment, viral status, CD8+ infiltration, specimen timing, assay, scored compartment, and spatial architecture.

## 6. MCC-Specific Clinical Guidance, Regulatory Status, and Treatment Evidence

### 6.1. Disease-Specific Guidance and Jurisdiction-Specific Authorizations

MCC-specific clinical guidance should be distinguished from general PD-L1 assay guidance and regulatory authorization. The European Society for Medical Oncology (ESMO)–European Reference Network for Rare Adult Solid Cancers (EURACAN) guideline addresses MCC diagnosis, staging, local treatment, and systemic management and supports PD-1/PD-L1 blockade for inoperable or metastatic disease [19]. The professional National Comprehensive Cancer Network (NCCN) guideline examined here is Version 2.2026, dated 24 October 2025 [94]. The National Cancer Institute PDQ is a complementary treatment-evidence summary, not a formal guideline or policy statement [95]. Pooled advanced-disease studies report an objective response rate of approximately 54%, with more durable control than historical chemotherapy, but do not establish comparative efficacy between agents [96,97].

The NCCN systemic-therapy algorithms list avelumab, nivolumab, pembrolizumab, and retifanlimab as preferred regimens for regional disease when curative surgery and curative radiotherapy are not feasible, and for disseminated M1 disease. Neoadjuvant nivolumab is an option in selected primary locally advanced or regional presentations. Ipilimumab plus nivolumab is listed under “Other Recommended”, with particular relevance to disease refractory to PD-1 therapy. These recommendations are organized by clinical setting, not a PD-L1 immunohistochemical threshold, and guideline inclusion must not be equated with a specific FDA or EMA authorization. NCCN Version 2.2026 does not support routine adjuvant immunotherapy outside a clinical trial [94].

The following jurisdiction-specific indications reflect the regulatory information assessed on 6 September 2026. In the United States, avelumab is indicated for adults and patients aged 12 years or older with metastatic MCC [98]; pembrolizumab is indicated for adult and pediatric patients with recurrent locally advanced or metastatic MCC [99], with the regulatory basis summarized in a peer-reviewed FDA approval review [100]; and retifanlimab is indicated for adults with metastatic or recurrent locally advanced MCC [101], following accelerated approval in March 2023 [102]. In the European Union, avelumab is authorized for adults with metastatic MCC and retifanlimab is authorized as first-line monotherapy for adults with metastatic or recurrent locally advanced MCC not amenable to curative surgery or radiation; the Keytruda product information examined at that date did not list an MCC indication [103,104,105]. These jurisdiction-specific labels do not require PD-L1 testing or a positivity cutoff for MCC. Therefore, PD-L1-negative staining must not be used to deny otherwise appropriate PD-1/PD-L1 blockade. Table 4 summarizes the regulatory status, guideline-supported uses, and principal clinical evidence for immune checkpoint therapies in MCC, together with the role of PD-L1 testing in each setting.

### 6.2. Principal Therapeutic Evidence and the Perioperative Setting

Avelumab is a fully human IgG1 anti-PD-L1 antibody. Long-term follow-up of previously treated metastatic MCC demonstrated durable responses, and first-line cohorts confirmed clinically meaningful activity [52,77,106,107]. Its Fc region can mediate antibody-dependent cellular cytotoxicity in experimental systems, although the clinical contribution of this mechanism in MCC remains uncertain [118]. Pembrolizumab, an IgG4 anti-PD-1 antibody, produced objective responses in approximately one half of previously untreated patients with advanced MCC, with durable benefit on extended follow-up and in KEYNOTE-913 [51,76,108,109]. Retifanlimab, another anti-PD-1 antibody, achieved an objective response rate of approximately 52–55% in POD1UM-201 and is administered every 4 weeks [54]. Cross-trial numerical comparisons are inappropriate because eligibility, treatment line, follow-up, and endpoint definitions differ.

Selected neoadjuvant use should be distinguished from routine perioperative treatment. NCCN includes neoadjuvant nivolumab in specific clinical scenarios [94], and CheckMate 358 reported pathologic complete response in 17/36 resected patients (47.2%) [110]. In a subsequent single-center phase II study of lenvatinib plus pembrolizumab, pCR occurred in 15/26 patients (57.7%), radiographic response in 16/22 evaluable patients (72.7%), and grade 3 treatment-related adverse events in 14/26 (53.8%), without grade 4–5 events [112]. Different endpoint denominators and nonrandomized designs preclude direct efficacy comparisons; the combination data do not establish a routine neoadjuvant regimen or a PD-L1-based selection rule.

ADMEC-O showed a statistically inconclusive disease-free-survival signal for adjuvant nivolumab versus observation (hazard ratio 0.58, 95% confidence interval 0.30–1.12), with greater toxicity [111]. Two National Cancer Database analyses associated combined immunotherapy and radiotherapy with favorable survival, but possible cohort overlap, treatment selection, and residual confounding prevent causal inference [113,114]. ADAM (NCT03271372) reported a stratified RFS hazard ratio of 0.61 (95% confidence interval 0.32–1.16); neither this nor the prespecified adjusted analysis met the conventional two-sided 0.05 threshold [115,116].

The updated STAMP/EA6174 abstract reported overall relapse-free survival (RFS; recurrence or death from any cause) with a one-sided *p* = 0.10. Distant metastasis-free survival (distant recurrence or MCC-related death) favored pembrolizumab at one-sided *p* = 0.05, as did MCC-specific RFS (MCC recurrence or MCC-related death; hazard ratio 0.66, 90% confidence interval 0.45–0.96; one-sided *p* = 0.032) [117]. These MCC-specific analyses do not replace the unmet overall RFS endpoint in the prespecified hierarchy of RFS followed by overall survival and do not establish a perioperative regulatory indication or a PD-L1 selection rule.

Nivolumab plus ipilimumab has also been studied in ICI-naïve advanced disease [53,55]; activity after prior PD-1/PD-L1 therapy is supported directly by Kim et al. [119]. The latter setting must be distinguished from first-line single-agent treatment, particularly given combination toxicity.

## 7. Proposed Pathology Reporting Framework

The NCCN pathology principles support synoptic tumor reporting and description of the lymphocytic infiltrate, but do not list PD-L1 as a required reporting element in MCC [94]. Nevertheless, PD-L1 staining may be requested for research, multidisciplinary discussion, or other clearly defined purposes. Figure 2 summarizes a proposed, non-validated workflow linking diagnostic confirmation, selective PD-L1 assessment, and integrated pathology reporting.

An accurate diagnosis is the essential starting point. Following clinical suspicion, histological assessment and appropriate immunohistochemistry should confirm MCC and exclude mimics. If PD-L1 assessment is not requested, routine pathology reporting and standard management proceed without it. When testing is requested, its purpose and the specimen characteristics should be documented, including whether the sample represents a primary tumor or metastasis and whether treatment has already been administered. Technical adequacy should be assessed according to the assay used, considering viable tumor content, limited sampling, necrosis, inflammation, and tissue heterogeneity. The antibody clone and staining platform should also be recorded. If adequacy criteria are not met, the result should be reported as “not assessable” rather than “negative,” with the reason stated and no numerical staining estimate provided.

PD-L1 expression in tumor and immune cells should be assessed and described separately, with attention to spatial distribution and the scoring method used. The TPS, IC, and CPS approaches illustrated in Figure 2 are not mandatory scoring systems for MCC; in particular, IC scoring requires an assay-specific definition. These findings should be incorporated into a report that documents the specimen, assay, compartment-specific staining, scoring method, and interpretive limitations.

For an adequate specimen, a concise report may state: “PD-L1 immunohistochemistry (clone/platform) shows membranous staining in approximately X% of viable tumor cells. Immune-cell PD-L1 staining: [description of extent and spatial distribution]. No clinically validated PD-L1 cutoff or approved companion diagnostic is available for MCC; this result does not determine eligibility for immune checkpoint therapy.” Interpretation should consider MCPyV status and CD8+ T-cell infiltration when available, together with the clinical setting. The clinical-context panel in Figure 2 provides an overview of management settings rather than a treatment pathway determined by PD-L1 staining. This approach reflects broader pathology experience, in which PD-L1 interpretation depends on the tumor type, assay, cellular compartment, and clinical context [86,88,90,91,120].

## 8. Resistance Mechanisms and Emerging Biomarkers Beyond PD-L1

Primary and acquired resistance show why a single surface marker is inadequate. Loss or suppression of HLA class I antigen presentation can prevent tumor recognition even when PD-L1 is present. Multi-omic studies have demonstrated reversible viral and epigenetic repression of HLA class I pathways in MCC, while earlier work showed that reduced MHC class I expression is common and can be restored by interferon exposure [78,79]. These defects may uncouple an apparently inflamed microenvironment from effective tumor-cell killing.

Cross-tumor studies identify PD-1+TCF1+ stem-like or precursor-exhausted CD8+ T-cells as a proliferative source after checkpoint blockade, whereas terminally exhausted populations are less readily reinvigorated [121]. In MCC, central-memory T-cell architecture and T-cell receptor diversity have been associated with response [56,58], and tumor-infiltrating lymphocytes may co-express LAG-3, TIM-3, TIGIT, and CTLA-4 [37,40,41,42,43,70]. Broader literature provides mechanistic context for TIM-3 [122]. The favorable invasive-front FOXP3-low-cell association described in Section 5.1 must be distinguished from tumor-cell TIGIT expression, which showed only a nonsignificant favorable survival trend in Iwasaki’s study (*p* = 0.1680) [41]. Circulating PD-1+ TIGIT+ CD8+ cells represent a different compartment and have been associated with response to anti-PD-1 therapy [43]. None of these findings validates a routine MCC treatment-selection test.

Myeloid and innate compartments provide additional resistance routes. MCC-specific work has linked suppressive myeloid-cell populations with resistance to PD-1 blockade [47]. Broader tumor-immunology literature implicates dendritic-cell dysfunction, suppressive cytokines, metabolic competition, and hypoxia in limiting effective CD8+ immunity [123]. The inverse relationship between G6PD and PD-L1/immune activity provides an exploratory metabolic correlate of immune exclusion in MCC [72]. Acquired resistance after adoptive T-cell therapy has been linked to transcriptional loss of class I HLA [80]. Experimental epigenetic priming can restore components of the HLA class I processing machinery and MICA/MICB expression, suggesting potentially reversible immune escape [124,125]. These findings support combination strategies but remain translational rather than validated pathology biomarkers.

Clinical evidence after progression on anti-PD-(L)1 therapy remains limited but is more mature than single-case experience. In an international retrospective cohort of 185 patients, 108 developed progressive disease. Response data for subsequent systemic therapy, with or without additional local treatment, were available for 50 patients. Among them, 18 received platinum-doublet chemotherapy, with an objective response rate of 67% and a median response duration of 5.0 months; 16 underwent anti-PD-(L)1 rechallenge, with an objective response rate of 56% and a median response duration of 20.2 months. Selection bias, heterogeneous definitions of resistance, and treatment heterogeneity preclude direct comparative inference [126]. A separate Seattle-based retrospective cohort restricted to patients who progressed during or within 12 weeks of prior anti-PD-(L)1 therapy found only one partial response among 16 patients re-exposed to single-agent anti-PD-(L)1 (objective response rate 6%, 95% confidence interval 0.2–30%), with a median progression-free survival of 2.2 months [127]. Differences in eligibility, resistance definitions, and treatment composition, including the use of additional local treatment, make the two rechallenge cohorts non-equivalent; they should not be interpreted as a comparison of two monotherapy populations. The more strictly refractory series argues against routine single-agent same-class re-exposure after confirmed resistance. In the randomized phase II MATRiX trial, tuvusertib monotherapy met prespecified futility criteria without objective responses, whereas tuvusertib plus avelumab produced an objective response rate of 20.8% (95% confidence interval 7.1–42.2%) among 24 eligible patients with anti-PD-(L)1-refractory MCC. Responses occurred in both primary and acquired resistance. Treatment-emergent adverse events of grade 3 or higher occurred in 80% of patients receiving tuvusertib alone and 58% receiving the combination; the cohort was small, and these data require confirmation in larger series [128].

Future biomarker studies should also preserve the distinction between MCPyV-positive and MCPyV-negative MCC. These molecular groups differ not only in antigen source and mutational burden, but also in morphology and immunophenotype [81]. An integrated model may ultimately combine viral status, CD8+ density and proximity, tertiary lymphoid structures, HLA competence, suppressive myeloid populations, and alternative checkpoints; at present, no composite classifier has sufficient prospective validation for routine use.

## 9. Future Directions

Progress requires moving from retrospective single-marker studies to prospectively annotated, multicenter tissue cohorts. Essential design features include prespecified specimen timing, separate analysis of primary tumors and metastases, whole-slide assessment when feasible, centralized or validated local staining, explicit clone/platform documentation, reproducible tumor- and immune-cell segmentation, and blinded outcome analysis. Studies should report continuous distributions before exploring cutoffs and should validate any threshold in an independent cohort.

Multiplex immunohistochemistry, digital image analysis, spatial transcriptomics, and T-cell receptor profiling can test whether immune-cell identity and geographic interaction outperform PD-L1 percentage alone. Clinically useful models will need to demonstrate incremental value beyond stage, performance status, disease burden, treatment line, and viral status. Equally important, biomarker development should not delay treatment in patients who already meet clinical criteria for approved PD-1/PD-L1 blockade. More broadly, the expanding use of next-generation sequencing in cutaneous pathology may facilitate integrated MCC assessment of viral status, mutational profiles, and immune-escape alterations [22,26,27,28,78,129].

## 10. Limitations

As a narrative review, this synthesis is selective rather than exhaustive. Abstract-only reporting, missing assay specifications, incomplete biomarker data, and reliance on imputation constrain parts of the evidence base. Most MCC PD-L1 studies are small retrospective series with heterogeneous sampling, treatment, clones, compartments, cutoffs, and outcomes; some institutional cohorts overlap. Secondary synthesis cannot supply independent replication when its MCC survival result derives from a single primary study. Publication, reporting, and selection biases remain possible. Associations among treated patients do not establish treatment-specific prediction, and multivariable adjustment does not replace independent validation. Cross-tumor and experimental-model findings remain contextual because their clinical applicability to MCC is uncertain.

Therapeutic evidence is frequently single-arm or modest in size, and cross-trial comparisons cannot establish relative efficacy. Perioperative studies include nonrandomized single-center cohorts, potentially overlapping observational database analyses, and conference reports requiring further confirmation. Regulatory labels and dated guideline versions may also change.

## 11. Conclusions

PD-L1 in MCC is best understood as a context-dependent and often spatial marker of tumor–immune interaction. It may reflect IFN-γ-driven adaptive immune resistance, but current evidence does not support a validated stand-alone prognostic classifier, companion diagnostic, or universal scoring cutoff. Responses to PD-1/PD-L1 blockade occur in PD-L1-positive and PD-L1-negative tumors; treatment eligibility should therefore follow MCC-specific clinical guidance and regulatory indications, not baseline PD-L1 staining.

When PD-L1 testing is requested, pathologists should report the specimen, clone/platform, tumor- and immune-cell compartments, a continuous staining estimate when technically assessable, spatial heterogeneity, and limitations. Future studies should evaluate integrated, spatially resolved biomarkers that combine PD-L1 with MCPyV status, CD8+ T-cell architecture, antigen-presentation competence, tertiary lymphoid structures, and suppressive myeloid populations. Until such models are prospectively validated, transparent descriptive reporting is more defensible than an unsupported binary result.

## Figures and Tables

**Figure 1:**
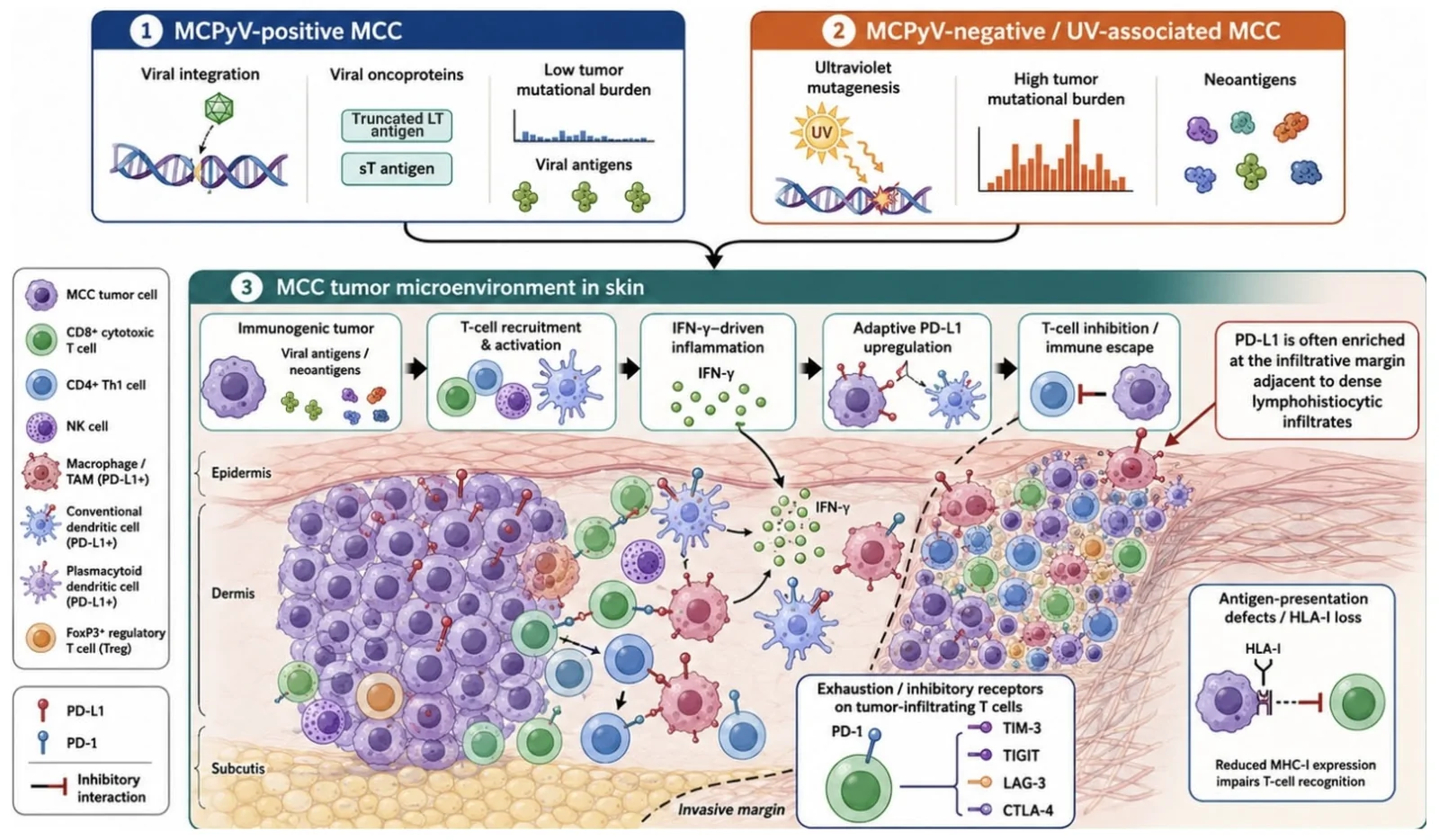
Conceptual immunopathological model of PD-L1 regulation in MCC. MCPyV-positive tumors express small T (sT) antigen and truncated large T (LT) antigen after viral integration; UV-associated tumors generate mutation-derived neoantigens. In both settings, lymphocyte recruitment and IFN-γ-driven inflammation can induce PD-L1 at tumor–immune interfaces. Engagement of PD-1 on T-cells by PD-L1 suppresses T-cell function. Alternative checkpoints and impaired antigen presentation may also limit tumor recognition. Solid black arrows indicate the direction of the depicted biological events or signalling relationships, including viral integration, UV-induced DNA damage, immune-cell recruitment/activation, IFN-γ production, and the sequential progression from tumor immunogenicity to adaptive PD-L1 upregulation and T-cell inhibition. The red arrow identifies the infiltrative tumor margin where PD-L1-positive cells are enriched adjacent to the lymphohistiocytic infiltrate. T-shaped bars indicate inhibitory interactions, whereas the dashed black line delineates the invasive tumor margin.

**Figure 2:**
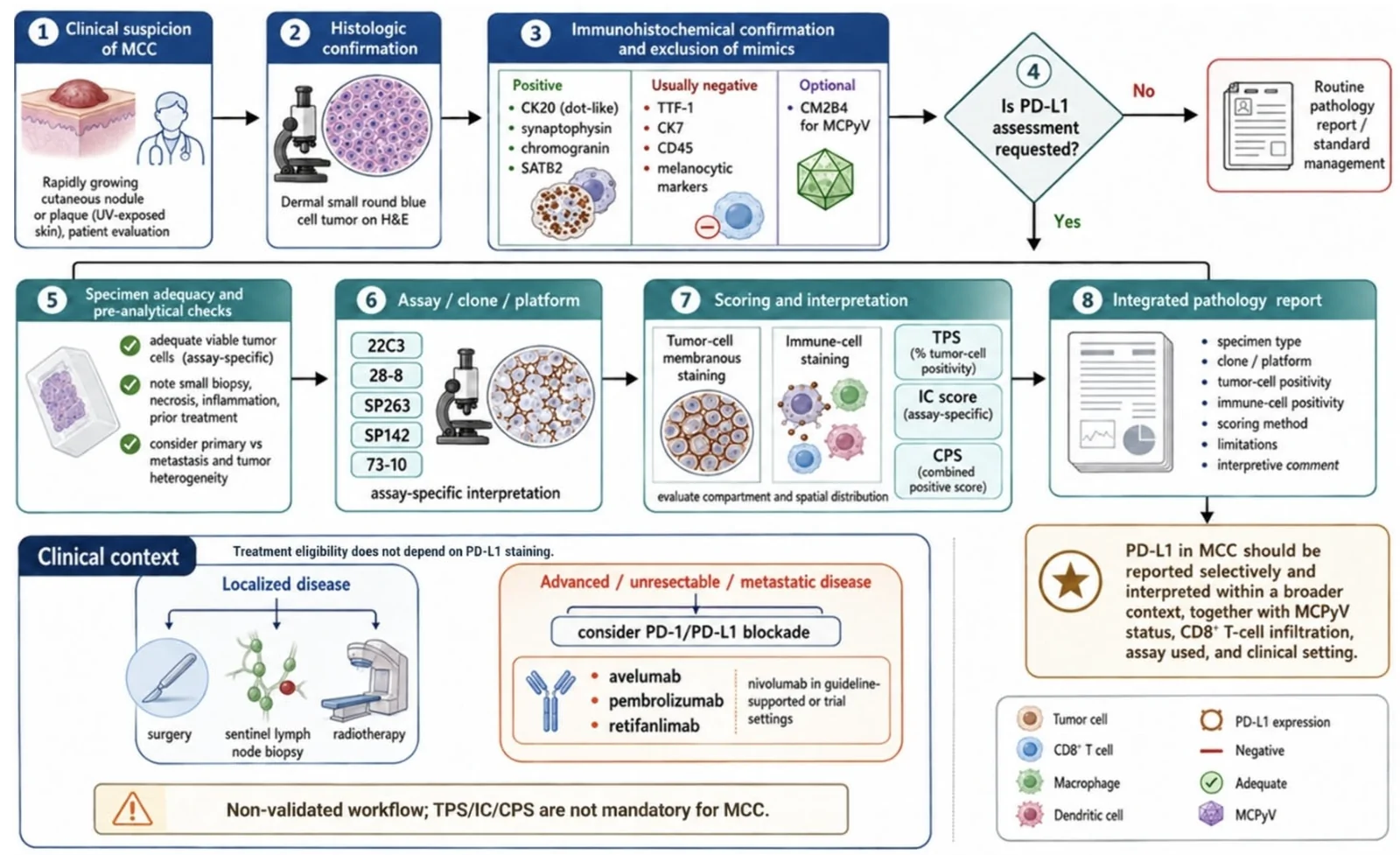
Suggested pathology workflow for selective PD-L1 assessment in MCC. Following diagnostic confirmation, the workflow outlines specimen assessment, assay documentation, compartment-specific interpretation, and reporting. Adequacy criteria and scoring methods are assay-specific, and treatment eligibility does not depend on PD-L1 staining. The proposed workflow has not been clinically validated and is intended to support descriptive reporting, not treatment selection.

**Table 1:** Evidence boundaries for interpreting PD-L1 in MCC.

Domain	Direct MCC Evidence	Current Interpretation	Practical Consequence
Biological meaning	PD-L1 occurs on tumor and immune cells and is frequently enriched near lymphohistiocytic infiltrates or at tumor borders [39,40,60,62,63,64].	Usually consistent with adaptive immune resistance, while constitutive or treatment-modified expression may also occur.	Interpret the compartment and spatial pattern, not only a percentage.
Prognosis	Direct cohorts report favorable, null, and non-independent associations; results differ between tumor-cell and immune-cell scoring [39,44,48,61,62,64,65,66,67,68,69,70,71].	No validated independent prognostic role.	Do not assign a prognostic risk class from PD-L1 alone.
Prediction of ICI response	Small spatial studies show associations, whereas larger cohorts and trials show responses across PD-L1 groups [51,52,56,75,76,77].	Binary PD-L1 status is insufficient for treatment selection.	Do not use PD-L1 negativity to exclude an otherwise eligible patient from ICI therapy.
Assay and cutoff	MCC studies use heterogeneous clones, compartments, and thresholds. MCC-specific clinical interchangeability of PD-L1 assays has not been established [39,44,48,60,61,75].	No clone, scoring algorithm, or cutoff is clinically mandated specifically for MCC.	Record the exact assay and avoid importing cutoffs from other tumors.
Integrated biomarkers	CD8+ density/proximity, viral status, tertiary lymphoid structures, HLA competence, myeloid populations, TIGIT, and G6PD show investigational value [34,41,47,56,57,70,72,74,78,79,80,81].	Composite spatial models are promising but unvalidated.	Restrict composite or integrated scoring to research until prospective validation.

Abbreviations: CD8, cluster of differentiation 8; G6PD, glucose-6-phosphate dehydrogenase; HLA, human leukocyte antigen; ICI, immune checkpoint inhibitor; MCC, Merkel cell carcinoma; PD-L1, programmed death ligand 1; TIGIT, T-cell immunoreceptor with immunoglobulin and immunoreceptor tyrosine-based inhibitory motif domains.

**Table 2:** Cross-tumor analytical evidence for major PD-L1 assays and its limited implication for MCC.

Assay/Clone	Original Context and Comparative Pattern	Boundary for MCC
22C3 pharmDx	Drug- and tumor-specific TPS/CPS; tumor-cell staining often aligns with 28-8/SP263 in NSCLC [88,89,90].	No MCC companion-diagnostic indication or validated treatment-selection cutoff.
28-8 pharmDx	Nivolumab development; broadly concordant tumor-cell staining with 22C3/SP263 [88,90].	Do not infer equivalence for immune-cell scoring.
SP263	Indication-specific protocols; tumor-cell staining generally clusters with 22C3/28-8 [88,90].	Document platform and locally validated protocol.
SP142	Often immune-cell algorithms; lower tumor-cell sensitivity in comparative studies [90].	Unsupported assay substitution can change positivity rates.
73-10	Avelumab development; higher tumor-cell analytical sensitivity in Blueprint comparisons [90,91].	Analytical sensitivity does not establish an MCC predictive cutoff.

The analytical comparisons summarized here derive predominantly from non-small cell lung cancer and do not establish MCC-specific clinical interchangeability. Abbreviations: CPS, combined positive score; MCC, Merkel cell carcinoma; NSCLC, non-small cell lung cancer; PD-L1, programmed death ligand 1; TPS, tumor proportion score.

**Table 3:** Selected primary MCC studies compared by sampling, assay, scoring compartment, endpoint, and adjustment.

Study/Evaluable Material	Specimen and PD-L1 Assay	Compartment/Threshold	Endpoint and Principal Result	Adjustment/Interpretive Limit
Lipson [39] 49 patients	Archival FFPE sections; clone 5H1.	Tumor-cell membranous staining ≥ 5%; immune context assessed separately.	PD-L1 absence vs. presence: OS HR 3.12 (95% CI 1.28–7.61), *p* = 0.012.	Adjusted for age and anatomical site; retrospective, single center.
Bénigni [60] 58 tumors	Serial 3 μm FFPE whole sections; 22C3 (Dako, 1:50), BenchMark Ultra/OptiView DAB with amplification.	TC membrane ≥ 1% on whole slide; selected positive fields also required ≥ 1% stained TC.	11/58 positive by whole-slide criterion; focal positive fields in 22/47 remaining tumors.	Spatial sampling only; no ICI or clinical-outcome validation.
Hanna [44] 78 patients; 67 PD-L1-evaluable	Triplicate tissue microarrays; clone 73-10. No ICI treatment.	Tumor-cell H-score > 0, rather than a fixed percentage cutoff.	No significant OS association: HR 1.26 (95% CI 0.46–3.45), *p* = 0.60.	Retrospective; limited events and heterogeneous disease sites; TMA sampling.
Guénolé [68] 77 non-metastatic patients; 58 primary tumors with IHC	Whole FFPE sections; 22C3 (Dako, 1:50), BenchMark Ultra/OptiView DAB with amplification; no ICI treatment.	TC and IC membrane staining assessed separately; ≥1% vs. <1% in each compartment. TC-positive 11/58; IC-positive 40/58.	OS: TC univariate HR 0.39 (0.18–0.83), adjusted 0.82 (0.31–2.15; *p* = 0.69). IC adjusted HR 0.43 (0.20–0.79; *p* = 0.006); also favorable regional control and DMFS.	Surgery ± radiotherapy; independent association concerns IC, not TC. Potential institutional overlap with Bénigni; independence is not established.
Donizy [48] 132 primary tumors	Two 2 mm TMA cores; E1L3N, Leica Bond 3.	Tumor-cell membranous staining > 1%.	Favorable univariate OS, MCC-specific survival, and PFS; no independent multivariable PD-L1 effect.	Primary tumors only; correlated immune variables and multiple endpoints.
Siqueira [61] 65 patients	Primary tissue preferred; metastasis if primary unavailable; SP263, Ventana.	Tumor-cell thresholds ≥ 1% and ≥5%.	Association with MCPyV, but no significant PFS or OS association.	Retrospective single-center cohort; mixed sites and clinical stages.
Toberer [70] 21 primaries + 6 metastases; DSS analysis in 21 primary cases	Entire FFPE sections; SP263, Ventana BenchMark Ultra.	Tumor-cell TPS and immune-cell score assessed separately; positivity ≥ 1%.	Immune-cell positivity associated with longer DSS (log-rank *p* = 0.011).	No multivariable survival validation; small cohort and multiple comparisons.
Comut [71] 35-case multicenter series	PD-L1 CPS evaluated with PRAME, MCPyV, and TILs; PD-L1 clone and evaluable n unavailable in the consulted abstract.	CPS < 1 vs. ≥1.	CPS < 1 associated with shorter OS in the reported multivariable analysis.	Abstract-only evidence (full-text not available at the time of our search); 35 is the total cohort, not a verified PD-L1-evaluable denominator. Small retrospective series; independent validation required.
Giraldo [75] 26 patients; PD-L1 *n* = 25; PD-1 *n* = 16	Pretreatment whole sections; PD-L1 clone 22C3; PD-1 immunofluorescence.	TC, IC, and combined positivity at ≥1%; digital positive-cell density (cells/mm^2^), PD-L1-positive tissue area, and PD-1/PD-L1 proximity within 20 μm.	PD-L1 density/area and PD-1/PD-L1 proximity associated with pembrolizumab response; compartment-specific binary scores were not associated.	Nested analysis of the initial KEYNOTE-017 cohort, not independent clinical replication; small assay-specific subsets; no validated clinical threshold.
Spassova [56] 114 clinical patients; PD-L1 observed *n* = 38; separate multiplex *n* = 21	Recorded tumor PD-L1 status; antibody clone not specified. Separate pretreatment multiplex panels did not include PD-L1.	Binary tumor PD-L1: 21 positive, 17 negative; cutoff not specified in main text/supplements. Multiplex: 11 disease-control vs. 10 progression cases.	No relevant PD-L1 association in ordinal BOR model. Separate T-cell phenotype/proximity findings concerned disease control (CR/PR/SD).	76/114 PD-L1 results missing; multiple imputation (30 datasets), not a 38-case complete-case model. 41 patients previously reported [58].
Schielke [93] 39 advanced MCC patients	Pretreatment FFPE; 28-8 pharmDx, Dako Autostainer Link 48.	Tumor-cell membrane ≥ 1%; ≥100 assessable tumor cells.	Adjusted OS HR 0.35 (95% CI 0.14–0.90). Responses: 10/23 positive vs. 4/16 negative; difference not significant.	Retrospective single center; mixed agents/lines and exploratory models; not predictive validation.
Secondary synthesis Abdollahi [92] MCC OS: one study (Guénolé); 77 clinical/58 IHC cases	Skin-neoplasm meta-analysis: 24 studies overall; four MCC reports listed, only one contributing to MCC OS.	Guénolé TC staining ≥ 1% vs. <1%; 22C3 on BenchMark Ultra [68].	MCC OS HR 0.39 (95% CI 0.18–0.83), reproducing Guénolé’s univariate TC result.	Single unadjusted source contrast; no multicohort MCC pooling or independent confirmation. The source TC effect did not persist after adjustment.

Intervals accompanying HRs are 95% confidence intervals. Contrasts and endpoints differ; no new pooling is performed. Bénigni and Guénolé may share institutional material and are not assigned a combined independent sample size. Abbreviations: BOR, best overall response; CI, confidence interval; CPS, combined positive score; CR, complete response; DMFS, distant metastasis-free survival; DSS, disease-specific survival; FFPE, formalin-fixed paraffin-embedded; HR, hazard ratio; IC, immune cells; ICI, immune checkpoint inhibitor; IHC, immunohistochemistry; MCC, Merkel cell carcinoma; MCPyV, Merkel cell polyomavirus; OS, overall survival; PD-1, programmed cell death protein 1; PD-L1, programmed death ligand 1; PFS, progression-free survival; PR, partial response; PRAME, preferentially expressed antigen in melanoma; SD, stable disease; TC, tumor cells; TMA, tissue microarray; TPS, tumor proportion score.

**Table 4:** MCC-specific regulatory and clinical context.

Agent or Treatment Strategy	MCC-Specific Regulatory Status	Principal Evidence	PD-L1 Requirement
Avelumab	FDA: adults and pediatric patients aged 12 years or older with metastatic MCC; EMA: adults with metastatic MCC [98,103].	JAVELIN Merkel 200, previously treated and first-line cohorts [52,77,106,107]. PD-L1-stratified first-line results and assay details are discussed in Section 5.2.	Not required.
Pembrolizumab	FDA: adult and pediatric patients with recurrent locally advanced or metastatic MCC; no current EMA indication for MCC [99,100,104].	CITN-09/KEYNOTE-017 and KEYNOTE-913 [51,76,108,109].	Not required for the FDA MCC indication.
Retifanlimab	FDA: adults with metastatic or recurrent locally advanced MCC. EMA: first-line monotherapy in adults with metastatic or recurrent locally advanced MCC not amenable to curative surgery or radiotherapy [101,102,105].	POD1UM-201 [54].	Not required.
Nivolumab	No specific FDA or EMA MCC indication. NCCN supports monotherapy in selected advanced settings and selected neoadjuvant use [94].	Advanced monotherapy/combination studies, neoadjuvant CheckMate 358, and adjuvant ADMEC-O [53,55,110,111].	No validated PD-L1 threshold for the NCCN treatment recommendations [94].
Pembrolizumab + lenvatinib (neoadjuvant)	No neoadjuvant FDA or EMA MCC indication.	Single-center phase II: pCR 15/26 (57.7%); radiographic ORR 16/22 (72.7%); grade 3 TRAEs 14/26 (53.8%); no grade 4–5 TRAEs [112].	No validated role for PD-L1 selection.
Adjuvant immunotherapy ± radiotherapy (real-world)	No adjuvant FDA or EMA MCC indication.	Two NCDB analyses: no consistent overall-survival benefit for immunotherapy alone; favorable associations with immunotherapy plus radiotherapy. Observational data with possible cohort overlap [113,114].	No PD-L1-based treatment-selection conclusion can be drawn from these analyses.
Avelumab (adjuvant)	No adjuvant FDA or EMA MCC indication.	ADAM registration and phase III report: neither the stratified nor the prespecified adjusted stratified RFS comparison met the conventional two-sided 0.05 threshold [115,116].	No validated role for PD-L1 selection.
Pembrolizumab (adjuvant)	No adjuvant FDA or EMA MCC indication.	STAMP/EA6174 phase III abstract: overall RFS *p* = 0.10; DMFS *p* = 0.05; MCC-specific RFS HR 0.66 (90% CI 0.45–0.96), *p* = 0.032. All *p*-values one-sided; overall RFS endpoint not met [117].	No validated role for PD-L1 selection.

Abbreviations: CI, confidence interval; DMFS, distant metastasis-free survival; EMA, European Medicines Agency; FDA, U.S. Food and Drug Administration; HR, hazard ratio; MCC, Merkel cell carcinoma; NCCN, National Comprehensive Cancer Network; NCDB, National Cancer Database; ORR, objective response rate; pCR, pathologic complete response; PD-L1, programmed death ligand 1; RFS, relapse-free survival; TRAE, treatment-related adverse event.

## Data Availability

No new data were created or analyzed in this study. Data sharing is not applicable to this article.
